# Supplementary material for: Content-rich biological network constructed by mining PubMed abstracts
Source: BMC Bioinformatics. 2004 Oct 8;5:147. doi: 10.1186/1471-2105-5-147 (PMC528731; doi:10.1186/1471-2105-5-147)
Supplement: Additional File 5 — The original Chilibot query results of the term "long-term potentiation (LTP)" and 22 other terms, limiting the latest references analyzed to the years 1990, 1995, 2000, and 2004. [file 1471-2105-5-147-S5.bz2 › chilibotAdditionalFile5/ltp2000/html/CAMKII_ACTININ.html]

 


 **CAMKII** and **ACTININ** 
  
Found 1 abstracts in PubMed,  **1 abstracts were retrieved and analyzed**.  


---

 Search Google  |
 PDF files only 
|  EDU domain only 

---

- J Neurosci, 2000   **Postsynaptic scaffolds of excitatory and inhibitory synapses in hippocampal neurons maintenance of core components independent of actin filaments and microtubules.** .
  The mechanisms responsible for anchoring molecular components of postsynaptic specializations in the mammalian brain are not well understood but are presumed to involve associations with cytoskeletal elements.
  Here we build on previous studies of neurotransmitter receptors Allison et al.
  , 1998 to analyze the modes of attachment of scaffolding and signal transducing proteins of both glutamate and GABA postsynaptic sites to either the microtubule or microfilament cytoskeleton.
  Hippocampal pyramidal neurons in culture were treated with latrunculin A to depolymerize actin, with vincristine to depolymerize microtubules, or with Triton X 100 to extract soluble proteins.
  The synaptic clustering of PSD 95, a putative NMDA receptor anchoring protein and a core component of the postsynaptic density PSD , was unaffected by actin depolymerization, microtubule depolymerization, or detergent extraction.
  The same was largely true for GKAP, a PSD 95 interacting protein.
  In contrast, the synaptic clustering of Ca 2ion calmodulin dependent protein kinase II **CaMKII** alpha, another core component of the PSD, was completely dependent on an intact actin cytoskeleton and was partially disrupted by detergent.
  Drebrin and alpha **actinin** 2, actin binding proteins concentrated in spines, were also dependent on F actin for synaptic localization but were unaffected by detergent extraction.
  Surprisingly, the subcellular distributions of the inhibitory synaptic proteins GABA A R and gephyrin, which has a tubulin binding motif, were unaffected by depolymerization of microtubules or actin or by detergent extraction.
  These studies reveal an unsuspected heterogeneity in the modes of attachment of postsynaptic proteins to the cytoskeleton and support the idea that PSD 95 and gephyrin may be core scaffolding components independent of the actin or tubulin cytoskeleton.
